# Nutritional Health Risk (Food Security) in Thai Older Adults and Related Factors

**DOI:** 10.3390/nu16162703

**Published:** 2024-08-14

**Authors:** Teeranut Harnirattisai, Sararud Vuthiarpa, Lisa Renee Pawloski, Kevin Michael Curtin, Eden Blackwell, Jenny Nguyen, Sophia Madeleine Bourgeois

**Affiliations:** 1 Faculty of Nursing, Thammasat University, Khlong Nueng 12120, Thailand; teeranut@nurse.tu.ac.th; 2 Faculty of Nursing, Rattana Bundit University, Khlong Nueng 12160, Thailand; vsararud@yahoo.com; 3 Department of Anthropology, University of Alabama, Tuscaloosa, AL 35401, USA; beblackwell@crimson.ua.edu; 4 Department of Geography and the Environment, University of Alabama, Tuscaloosa, AL 35401, USA; kmcurtin@ua.edu; 5 Department of Biology, University of Alabama, Tuscaloosa, AL 35401, USA; jtnguyen2@crimson.ua.edu (J.N.); smbourgeois@crimson.ua.edu (S.M.B.)

**Keywords:** nutritional health risk, food security, Thai older adults

## Abstract

The older adult population in Thailand has been steadily increasing in recent years, and urbanization has resulted in many older adults living independently, leaving many at nutritional risk. The purpose of this research is to explore food security among Thai older adults using a simple screening tool, the DETERMINE tool, as well as from three surveys which reflect seniors’ health and ultimately food security including the mini-mental state examination (MMSE), the self-efficacy for physical activity scale (SEPAS), and the health literacy questionnaire. The DETERMINE tool was used in Thailand for the first time in this study. The findings revealed a moderate risk of food insecurity amongst participants, as most of them claimed to have underlying diseases, eat alone, eat a few nutrient-rich foods, and take medication. The MMSE, SEPAS, and health literacy questionnaire results suggested that food security was found to be negatively correlated with higher cognitive ability, higher physical activity, self-efficacy, and higher health literacy. In conclusion, there appears to be a high risk for malnutrition among older adults in Thailand, particularly in those with low income and underlying diseases.

## 1. Introduction

### 1.1. Overview of Food Security and Older Adults in Thailand

Nutritional health is an important component of independence and quality of life among older adults. Food security is a nutritional risk factor and necessary for good nutritional heath. Food security is defined by the World Health Organization as “when all people have physical, social, and economic access to sufficient, safe, and nutritious food that meets their dietary needs and food preferences” [1]. Food insecurity occurs when such access and availability is limited and can increase nutritional risk. Vulnerable populations at risk for food include but are not limited to those living in poverty, displaced peoples, children, and older adults. Older adult populations are often faced with limited finances, comorbidities, limited mobility, physiological changes, and other challenges that limit access to sufficient nutrient-dense foods. Thailand is not limited to such risk, particularly as the aging population in Thailand rises, and fewer resources become available to support the complexities of the aging needs. Phulkerd et al. [2] reported that Thailand holds the second highest number of individuals over 60 in the ASEAN member countries, and it has been projected that one in three Thais will be over the age of 60 years by 2040.

### 1.2. Nutrition and Food Security of Older Adults in Thailand

While obesity has increased dramatically among children and adolescents in Thailand over the past twenty years [3], it has also impacted older adults, increasing risk for chronic disease and disability. However, throughout Thailand there is still a greater risk for undernutrition among older adults, contributing to more complex health-related risks. Nawai et al. (2021) reported that in Chiang Mai Thailand, over 50% of older adults were malnourished (undernourished) or at risk of malnutrition [4]. These findings were greatly affected by socioeconomic status and functional status. Further, Vapattanawong et al. noted from a prospective study that older adults who were obese were actually protected more from mortality [5]. However, more recent literature suggests such protection is changing to a greater risk of obesity-related chronic diseases such as cardiovascular disease and diabetes. Duangjai et al. (2023) [6] recently reported that about 25% of their study population from a group of community-dwelling older adults (over 55 years) had a BMI over 25, and another study by Sukchan et al. (2022) [7] noted the prevalence of obesity among a group of older adults living in Southern Thailand was 35.2%. Obesity can also be associated with micronutrient deficiencies due to an overconsumption of calories but limited intake of nutrient-dense foods. Thus, when determining nutritional risk among older adult populations in Thailand, it is important to consider both under and over nutrition.

While there is growing literature reporting the prevalence and comorbidity relationships of nutritional status in older adults in Thailand, very few studies have examined its determinants, and particularly, the impact of food security on senior populations in Thailand. Much of the literature on the determinants of nutritional status include studies on food consumption patterns and selection and the impact of COVID-19. One recent study revealed that almost 30% of an aging population in Thailand exhibited indicators of food insecurity, which was also associated with financial hardships [8]. As food security is one of the major risk factors affecting nutritional health among older adults all over the world and food security in the Thailand literature is under-represented, particularly in older adults, we present an analysis using a simple tool to examine food insecurity among a population of older adults living in suburban Bangkok, Thailand. The purpose of this research is to explore food security among Thai older adults using a simple screening tool, the DETERMINE tool. In addition, we have presented data correlations with the findings from this tool from three other surveys which reflect senior health and ultimately food security, including cognitive function, physical activity self-efficacy, and health literacy.

### 1.3. Food Security Screening Tools and Use of DETERMINE Tool

As part of the US Nutrition Screening Initiative, a relatively simple to use, self-assessment tool was developed to determine nutrition and food security risk among older adults. This screening tool, “Determine Your Health Risk”, and noted in this paper as the DETERMINE tool, is also known as the Nutrition Screening Initiative checklist (NSI). The tool was developed in collaboration with the American Dietetic Association, the American Academy of Family Physicians, and the National Council on the Ageing [9]. The tool includes a checklist of ten questions to be answered yes or no. Each question is given a different weight that is associated with nutritional risk among the older adults. Nutritional risk includes poor calorie intake (too many or too few) and inadequate macronutrient and micronutrient intake. Nutrition risk can be impacted by food security, which includes access and availability to necessary nutrients. The DETERMINE tool can be helpful in predicting risk in community populations but is not intended to be a clinical diagnostic tool. As it is self-administered, it is helpful in assessing perceived health and nutritional risk; it may also help identify those with nutrient deficiencies [10]. The checklist has been validated in a few studies, and mostly in the U.S., a few studies have noted the tool to provide a weak predictor of mortality, but helpful in predicting nutrition risk [10,11].

In terms of international use, this tool has been used in studies in Denmark [10], Greece [12], and Europe more broadly [13,14], China [15], Singapore [16,17], and Japan [18,19], among others. These studies have shown usefulness in identifying and understanding the determinants of malnutrition and food insecurity among older adults. For example, one study found that food selection was based on foods that were “easy to chew”, thus reducing the variety and selection of nutrient-dense foods [20]. Further, one study identified a correlation with nutrient risk and fewer social interactions and food insecurity [12]. Many of these studies are conducted primarily to screen and identify malnourished older adults living in community settings. However, until now, the tool has not been used in Thai populations. Thus, this paper is the first to explore the findings from the DETERMINE tool in a Thai senior population. Here we present our findings on food security risk among Thai older adults using the DETERMINE tool, along with the results of a demographic survey of personal, familial, and basic health information. Those demographic factors that can be associated with higher nutritional health risk are explored vis a vis the cultural nutritional norms in Thailand.

### 1.4. Food Security in Asia

In Asia, the proportion of elders in the population is rapidly growing, and it is predicted that Asia will be the region with the largest older adult population in the world, exceeding over 4.9 billion (Asian Development Bank 2022). With this growing population, exploring the major issues that an aging society will face may be relevant for developing future interventions and policies. One of these issues is the widespread prevalence of malnutrition in older adult populations. Several studies have been conducted in Asian countries including China, Japan, Singapore, and Taiwan to assess nutritional risk and risk factors among older adults using the DETERMINE tool. Yap et al. (2007) and Sugiura et al. (2016) [17,19] assessed community-dwelling elders in Singapore and Japan, respectively, and found that activity of daily living disabilities and depression were significant indicators of high risk for poor nutrition using the DETERMINE tool. Sugiura [19] also found that declining functional capacity over a 2-year period was linked with higher nutritional risk. While functional disabilities and mental illness affect nutritional risk in elders, financial and educational barriers also play a role. Koo et al. (2013) [16] assessed the nutritional risk of senior recipients of financial assistance aged 55 and found that 50.3% were at risk of malnutrition. Major risk factors included advanced age, financial limitations, and lack of education about subsidized food sources. Understanding the determinants that influence nutritional risk and malnutrition in Asian elders may be beneficial for identifying at-risk populations and implementing the proper policies.

## 2. Materials and Methods

### Study Design and Study Population

This study was a cross-sectional study with the objective of surveying nutritional health risk in Thai older adults and related factors. The population group to be studied consisted of older adults (defined as 60 years of age or over) in the urban areas of two provinces of Thailand (Greater Bangkok Metropolitan Region). The goal was to conduct a representative survey using a multistage, stratified sampling of the Thai population. A demographic and lifestyle survey was used to collect information about the subjects’ age and gender, marital status, education, religion, and other family status variables. Information about underlying disease, smoking, and the use of alcohol was collected. The DETERMINE checklist was used as an indicator of nutritional health risk. Additional instruments were used to measure cognitive ability (mini mental state examination MMSE), and self-efficacy for physical activity (SEPAS), along with a health literacy questionnaire. SPSS software program version 22 was used to analyze the data. Statistics included descriptive statistics of the demographic data, with standard deviations noted for the interval ratio data. Frequencies and percentages were determined for the DETERMINE instrument for each item and means and standard deviations were calculated for each of the instruments. Finally, the Pearson Correlation Coefficient with a significance of *p* < 0.05 was used to examine the relationship between the tools.

For this study, the DETERMINE tool, also described earlier, was translated into Thai and back-translated to English with content experts in nutrition, geriatrics, and nursing. The sensitivity, specificity, and positive predictive value of the tool were originally conducted by Posner et al. (1993) [20] who evaluated the effectiveness of the tool and the nutritional risk cut-off points. The authors examined the reliability of the DETERMINE tool in Thailand using a random sampling with 30 participants and found Cronbach alpha of 0.7. The screening tool has been implemented in many Asian countries including Japan and Singapore, but this is the first use in Thailand.

More specifically, the mini-mental state examination test (MMSE-Thai 2002) of the Institute of Geriatric Medicine [21] is a set of questions for screening cognitive functions. This examination is suitable to indicate the presence of cognitive impairment, such as a person with dementia or suffering the effects of a head injury. The MMSE-Thai 2002 consists of 11 questions. Score interpretation is based on educational level. The instrument was validated for content validity by Thai content experts and was not retested. The reliability was validated through a coefficient by testing MMSE on 30 people with similar characteristics of the sample and the correlation coefficient was calculated to be 0.88. The maximum score for the MMSE survey is 30, and a score of 25 or higher is within the normal range. If the score is below 24, it may indicate possible cognitive impairment [22]. The self-efficacy for physical activity scale (SEPAS) has individuals rate their perceived confidence in their ability to perform each specific physical activity (e.g., leisure time activity, household activity, and work-related activity). SEPAS was developed by modifying the Self-Efficacy for Exercise by Resnick & Jenkins, 2000 [23].. It was also based on the Physical Activity Scale for older adults that look at physical activity in terms of leisure time, household tasks, and work-related activities [24]. The SEPAS contains 17 items and employs a semantic differential scale with scores ranging from 0 (no confidence) to 10 (total confidence). This instrument had been approved for content validated by five Thai experts and tested for reliability with 30 Thai older adults, resulting in an Alpha Cronbach of 0.90. Health literacy (knowledge capacity) refers to the ability of individuals to gain access to, understand, and use information in ways which promote and maintain good health for themselves. The health literacy questionnaire for older adults) [25,26] was analyzed in this study and the test for reliability resulted in an Alpha Cronback of 0.96. This questionnaire contains 22 items across 4 sections: (1) access to information (6 items), (2) understanding health information (5 item), (3) evaluating and selecting information (6 items), and (4) applying information (5 items). This questionnaire is a rating scale ranging from 6 (highest) to 1 (lowest).

The appropriate sample size was calculated based on G*Power. The calculation for correlation used alpha = 0.5, with effect size 0.3. The test employed a power of 80%, confidence interval of 95%, and acceptable error of 5%. The required number of participants according to the calculation was 263 cases. With an expectation of a 10% drop-out rate, a minimum of 290 older adults would need to be recruited for this study. A multistage, random sampling method was used for the study sample selection. In the first stage, based on the community characteristics of the two provinces, all 20 districts were divided into two categories of urban and rural areas. Based on the National Statistical Office Report [27], urban refers to municipalities which are inhabited by at least 200 persons per square kilometer, whereas rural indicates any area with a population density lower than 200 persons per square kilometer. According to this definition, there are a total of 12 subdistricts that are considered urban areas. A random selection of health promotion centers in those urban areas was carried out for this study.

At the last stage, the older adult subject population was randomly selected using the criteria described below and invited to participate in the study. The proportion of the samples from urban areas was based on a representative population size in six locations. A total of 302 participants with complete questionnaires were accepted. The inclusion criteria (beyond age and urban area classification) were (1) the respondents must be able to help themselves assessed by Activities of Daily Living (ADL) measurement, and (2) the respondents must have no dementia as assessed by a category test (Set Test). The exclusion criteria were (1) any active health problem or psychiatric condition that interferes with the provision of information, and (2) any problem of hearing, vision, or the ability to communicate in Thai with the researcher. The discontinuation criteria were (1) being unable to provide 100% information, (2) experiencing severe disease symptoms or being admitted to a hospital, and (3) any request to withdraw from the research. A summary of the demographic characteristics of the population is provided in Table 1.

This study was approved by the Human Research Ethics Committee of Thammasat University (Science) (COA No.048/2021 approved on 18 May 2020) prior to data collection. All sub-district health promotion centers responsible for the older adults were also granted approval. The researchers provided information regarding the purpose of the study to the participants, and written informed consent was obtained from all the participants. Data were collected for three months from August 2022 to October 2022. The researchers collaborated with the contact nurses in the sub-district health-promoting centers who helped to coordinate the identification of potential participants for the study. In the data collection at the older adult clubs, the researcher instructed the participants regarding the questionnaires and read aloud each item of the questionnaires and asked the participants to rate each item by themselves. Approximately 45 min were required to complete the questionnaire.

## 3. Results

Table 1 provides descriptive data of the sample. Most were female (81.5%), married (46.7%), reported as having inadequate income (54.7%), and unemployed (57.6%) or retired (86.2%). Also, few were educated beyond secondary school, and the mean age was 69.05 years. Further, most reported having some kind of underlying disease (90%) of which the majority had hypertension. Very few reported smoking (8%) and most (82%) reported never drinking alcohol. Overall, from the DETERMINE tool, the participants showed an average score of moderate risk (4.84), where 0 to 2 is good, 3–5 is of moderate risk, and 6 and higher is of great risk for food insecurity and malnutrition.

Table 2 presents the individual items for the DETERMINE tool and the frequency and percentage of those who noted those items. From these results, three of the indicators are interesting to highlight, which include the two items with the highest frequencies and the one item with the lowest frequency: (1) I have tooth and mouth problems that makes it difficult to eat. (2) I eat alone most of the time and (3) I drink beer, liquor, or wine three times (almost every day). In terms of frequency of responses, most participants noted issues with tooth and mouth problems (46%) and that they eat alone (52%). Also, the item that received the lowest frequency response was the item asking about drinking alcohol (2.6%). We discuss these three findings in greater detail in the Discussion section as well as address issues related to the other indicators noted. Other items that had high percentages and frequencies included items about eating few fruits and vegetables (33.1%), taking three or more over the counter or prescribed drugs per day (40.7), and having an illness (45%). Other items that noted low percentages and frequencies included eating fewer than two meals per day (7.6%), not being able to physically shop, cook, and nourish oneself (8.3%), without the need for gaining or losing weight within 6 months (9.3%), and not having enough money to buy food (21.2%).

Table 3 presents the mean and standard deviations of the items from the MMSE, health literacy, SEPAS, and food security instruments. The MMSE survey resulted in a mean score of 25.4; this falls within the normal range, but is slightly low, noting the maximum score is 30. The results of the health literacy tool resulted in a mean of 4.56, where 6 is the highest possible value. The SEPAS tool resulted in a mean of 7.52, where 10 equates to the highest confidence in perception of physical activity ability. Again, the DETERMINE mean score of 4.84 suggests moderate risk of food insecurity.

Table 4 presents correlation data that examine the relationships between the DETERMINE tool score and three other instruments related to health and wellness among seniors. These instruments measure cognitive ability, physical activity self-efficacy, and health literacy. The significance level is set at 0.05, and the statistical method used was the Pearson correlation coefficient. Food security was negatively correlated with all three measures, indicating that higher cognitive ability, higher physical activity self-efficacy, and higher health literacy are associated with a lower risk of food insecurity. 

## 4. Discussion

This is the first study identified through our literature review to use the DETERMINE tool to assess risk for food insecurity and malnutrition among older adults in Thailand. These results show that there is indeed a risk for malnutrition among older adults in Thailand, particularly among those who have limited income and those with underlying diseases. Interestingly, the results highlighted three areas which may be more specific to Thailand and be impacted by Thai culture and values. These include the three items which were selected at higher frequencies: (1) I have tooth and mouth problems that makes it difficult to eat, (2) I eat alone most of the time, and (3) I drink beer, liquor, or wine three times (a day?) almost every day. Each of these factors suggests an interpretation that is rooted in Thai culture, particularly Thai foodways and family dynamics.

Thailand’s unique foodways and traditional values related to aging intersect in ways that can have a meaningful impact on the health of older adult Thais. Thailand is well known for its varied cuisine which makes heavy use of herbs and fresh fruits and vegetables with rice and seafood staples [28,29]. Thai food culture has shifted in recent decades to meet the demands of labor market changes and features street food and “public eating” of meals prepared outside the home (which frequently include higher amounts of meats, fat, and sugar) [29,30]. Nonetheless, meal sharing is a culturally valued aspect of eating, particularly within the family [29]. Like much of Asia, Thailand has a strong tradition of filial piety and care of aging elders by younger generations; however, the performance of these obligations is shifting and is largely being met through financial remittances and long-distance communication rather than traditional co-residence in multi-generational households [31,32]. Increasingly, older Thais are finding themselves living and eating alone or without their adult children, with consequences for their diet and wellbeing [2,33]. In a study of elder happiness in Thailand, Phulkerd et al. [2] found that older adults who reported having all their meals with family members had the highest probability of happiness of adults who shared fewer meals with family.

Thai healthcare is widely available due to long-standing national healthcare provision programs. This suggests that those with persistent tooth and mouth problems may be at such a disadvantage that they cannot access even the most widely available healthcare. Analysis of the Thai National Oral Health Survey by Kaewkamnerdpong et al. [34] showed that the leading cause of mouth problems was tooth loss and too few occlusal pairs (pairs of teeth that make contact when chewing). More than half (60.6%) of Thai adults between 60 and 74 reported difficulty chewing food. Hyposalivation, or dry mouth, while it is not specified in the DETERMINE “mouth problems” item, is common in older age and has been linked to poor oral health, altered taste of food, and malnutrition [35]. Tooth loss and dry mouth contribute to poor chewing ability which frequently lead to avoiding certain foods, eating less, and ultimately poor diet. As noted above, adults in this study frequently reported low consumption of fruits and vegetables which require chewing or additional preparation to make them easier to consume for people with compromised ability to chew or swallow.

With regard to eating alone, although in rural populations there has been an increasing incidence of older people living alone while other, younger family members migrate to larger cities for employment opportunities, this is less prevalent in the urban and suburban areas where this study was conducted. Our study population reflects the national statistics in living arrangements for older adults in that most are aging at home with a spouse or other relatives, usually their adult children [36]. Therefore, this factor again suggests that those with greater health risk are the most vulnerable given their unusual lack of a familial support system. Further, Thai-specific values of intergenerational co-residence and psychological wellbeing are important to consider with using the DETERMINE tool in Thailand as loneliness and depression could drive poor eating and limited dietary diversity. Nawai [4] identified living alone as one of several factors associated with nutrition risk among older adults in Thailand. Older adults living alone were found to have decreased dietary diversity [33]. In an earlier study focusing on community-dwelling older people, Chalermsri et al. [37] found loneliness to be an important determinant of food choice which is related to nutrient intake. Research has also shown negative psychological effects of frequent (four times or more per week) eating alone where commensal eating is valued. Mikami et al. [18] found an association between frequent eating alone and poor appetite among older adults in Japan. In both China and Thailand, researchers have found associations between eating alone and depression or unhappiness, particularly among women [15,38]. Yiengprugsawan et al. [38] suggest these outcomes are linked to a departure from cultural norms.

Finally, the use of alcohol in anything other than moderation is not widely culturally acceptable among Thais. Men in Thailand are far more likely to drink than women who are considered “culturally protected” from related outcomes; the sample here is heavily skewed towards women, so the low report of alcohol consumption might be a factor of gender [39,40]. However, as Knodel and Pothisiri [41] found, the likelihood of drinking decreased for both men and women 60 and older who lived with family. While there are conflicting data on how Thailand compares against regional averages in alcohol consumption, rates of drinking have increased in recent decades with links to related increases in risks of Non-Communicable Diseases (NCDs) and poor diet [40]. This provides another piece of evidence that those with the highest nutritional health risk are often among those furthest from the core of Thai cultural norms. It may also be important to note that the DETERMINE criteria for alcohol consumption reflect daily drinking behaviors. Increased risks for poor diet (decreased fruits and vegetables and increased fried foods) and NCDs were found among Thai adults who engaged in non-daily binge drinking (four or more drinks per occasion according). Binge-drinking behaviors that threaten food security may be missed by the DETERMINE tool [40].

The other indicators having high frequencies included eating few fruits and vegetables, having an illness, and taking three or more over the counter or prescribed drugs per day. Eating few fruits and vegetables can be related to one of the highlighted indicators, tooth decay as well as ability to breakdown fibrous foods, common in Thai cuisine [42]. As noted earlier, cuisine in Thailand has become more public and there are fewer fruits and vegetables included. Nawai et al. [4] have shown that it is common in Thailand to see illnesses or conditions which lead to changes in eating habits in Thai elders, particularly dyslipidemias and osteoarthritis as well as severe changes in mental and physical health and limitations that these chronic illnesses cause. Those that take three or more over the counter drugs may lead to nutritional risk as medications may impact the appetite due to nausea, changes in taste, or nutrient interactions. While healthcare costs are relatively low in Thailand, medications may come at an extra cost that may impact the ability to purchase food for some with very low incomes. This is particularly a concern in the U.S. context.

Other indicators having lower frequency responses in this population included eating fewer than two meals per day, not being able to physically shop, cook, and nourish oneself, without the need for gaining or losing weight, and not having enough money to buy food. Food is widely available and accessible in Thailand, again, now in public settings. Thus, the availability and accessibility to have fewer than two meals a day might not be as common. Family and social support are also important in Thailand and while the necessities to cook are not always available due to transportation, mobility, etc., many social supports are available to assist with those who have physical limitations. The indicator related to losing or gaining weight without need would need to be explored more, but the low-response percentage may be due to the lack of access to scales and regular weight screening, one limitation which was noted in the development of a protein energy malnutrition screening tool in residential homes in Thailand [43]. Lastly, the indicator concerning not having enough money to buy food was noted by 21% of the participants, a lower frequency, but not low enough to ignore. While food is relatively inexpensive, available, and accessible, in Thailand, older adults concerned about medications and not bringing in income can be more greatly impacted economically. Further analyses in this population should be carried out to understand who is at most risk.

The correlations with the tools concerning cognitive ability, physical activity self-efficacy, and health literacy revealed expected results such that those with poorer cognitive ability, confidence in physical activity, and health literacy are at higher risk for food security. One importance of including these tools in these analyses was to add important components which the DETERMINE tool does not include or focus on, but are known determinants of poor senior health. Earlier literature has shown that poor health literacy is associated with poor physical activity and nutritional status [44].

These tools in addition to the DETERMINE tool are quite helpful in understanding determinants of health among senior populations. These results also suggest that the DETERMINE tool may be useful on its own when needing a fairly fast and simple screening tool to assess nutritional risk among senior populations in Thailand. The other tools for understanding senior health more comprehensively are more complex and take much longer to administer.

The DETERMINE screening tool may also assist health nurses and healthcare professionals to understand what focus of health education may be needed in order to decrease nutritional health risk and improve health status. Nurses play an important role in health education emphasizing on dental care, self-care for their chronic illness, eating with friends and family, and choices of foods that are specific for older adults. However, it is also important that nurses and health professional are aware of the impact of social determinants as well as health literacy, cognitive ability, and confidence in physical activity when providing health education.

## 5. Limitations and Implications for Practice

There are several limitations of this study. First, this study was conducted in a limited geographic area of two provinces that are largely urban or suburban. These findings cannot be immediately extended to rural populations without further investigation. Moreover, each of the provinces in this study are in the greater Metropolitan Bangkok region of Thailand. The results may vary in other regions of Thailand, particularly where there are variations in cultural institutions (e.g., ethnicity, religion). Further, the use of the DETERMINE tool may not entirely capture the local cultural and dietary characteristics that affect food security and nutrition among Thai older adults.

One challenge of using tools such as DETERMINE in different cultures and settings is to ensure that the items being used have an impact and to understand that certain items may have a greater impact or weight in the analyses. While this tool has been shown to be easily administered and useful in its simplicity as well as helpful in identifying at risk older adults, it is important to note that use in different cultures and settings may impact the overall score or selection of its items.

One additional limitation, regarding all the surveys, includes the use of self-reported data, which can be subject to bias. In this study, participants may have different perceptions regarding their dietary habits, health conditions, or the frequency of medication use. 

Despite these limitations, the clear findings outlined above regarding the factors associated with higher health risk clearly suggest some potential practical approaches that may alleviate nutritional insecurity. Given that older adults living alone, and those who consume alcohol regularly are at greatest risk, individuals with these characteristics can be identified during any routine health screening and provided with nutritional support, education regarding nutritional choices, or references to community support agencies that can ameliorate nutritional risks.

As the DETERMINE tool is a useful and simple tool for Thai populations, it is critical that the next steps include validity analyses to better weigh individuals items in the DETERMINE screening tool. The tool’s applicability and relevance in the Thai context need further validation to ensure its accuracy and reliability. Further research would include exploring the causal relationships with the results from the other surveys to best understand senior health and the use of these instruments. Also important would include confounding factors, such as social support systems, economic status, and access to healthcare services, which might also play significant roles. And lastly, additional analyses on the oral health of older adults would be helpful to better understand the nutritional risk of this population.

## 6. Conclusions

This article explores results from the DETERMINE screening tool among a group of older adults in urban and semi-urban Thailand. It also explores the relationship with these findings and other health indicators among older adults. The findings revealed that there is a high risk for malnutrition among older adults in Thailand, particularly in those with low-income and underlying diseases. While the tool is useful and simple, more research is needed to explore its applicability and relevance within the Thai context.

## Figures and Tables

**Table 1:** Descriptive demographic data of the study sample older adult population (*n* = 302).

Item	Response	#/Avg/%		Item	Response	#/Avg/%
Age	57–87 years	69.05 ± 6.18		Underlying disease	Having underlying diseases Diabetes mellitus Hypertension Heart/coronary artery disease Pulmonary disease Psychiatric disease Orthopedic disease Gastrointestinal disease Cerebrovascular disease Dyslipidemia Cancer Systemic lupus erythematous Allergic rhinitis Thyroid Cataract Benign prostate hyperplasia Parkinson disease Chronic kidney disease Rheumatoid arthritis Hepatitis Anemia Muscle weakness	272 (90.1%) 73 (24.2%) 157 (52.0%) 39 (12.9%) 5 (1.7%) 1 (0.3%) 45 (14.9%) 10 (3.3%) 7 (2.3%) 85 (28.1%) 7 (2.31%) 2 (0.6%) 6 (2.0%) 6 (2.0%) 3 (1.0%) 4 (1.3%) 1 (0.3%) 4 (1.3%) 1 (0.3%) 1 (0.3%) 3 (1.0%) 1 (0.3%)
Gender	Male Female	56 (18.5%) 246 (81.5%)				
Marital status	Single Married Divorced/separated Widowed	40 (13.2%) 141 (46.7%) 26 (8.6%) 95 (31.5%)				
Education level	Unlettered Elementary school Secondary school Vocational certificate Bachelor’s degree Postgraduate	20 (6.6%) 158 (52.3%) 74 (24.5%) 15 (5.0%) 31 (10.3%) 4 (1.3%)				
Religion	Buddhism Christianity Islam Missing	296 (98.0%) 3 (1.0%) 2 (0.7%) 1 (0.3%)				
Occupation	Agriculturist Merchant Officialdom Employee Company employee Unemployed Others Retirement Health volunteer Personal business	5 (1.7%) 45 (14.9%) 2 (0.7%) 43 (14.2%) 4 (1.3%) 174 (57.6%) 29 (9.6%) 25 (86.2%) 3 (10.3%) 1 (3.5%)		Smoking	Often Sometimes Seldom Never Missing	9 (3.0%) 3 (1.0%) 14 (4.6%) 270 (89.4%) 6 (2.0%)
Personal Income	0–100,000	6,522.57 ± 10,159.51		Alcohol consumption	Often Sometimes Seldom Never Missing	3 (1.0%) 27 (8.9%) 21 (7.0%) 248 (82.1%) 3 (1.0%)
Family Income	0–200,000	16,549.11 ± 20,686.70				
Adequate income	Adequate Inadequate Saving Dept	94 (31.1%) 165 (54.7%) 23 (7.6%) 20 (6.6%)				
				Nutrition health risk score		4.84 ± 3.55
Caregiver	Couples Sibling Children Grandchild Neighbor Health volunteer Formal caregiver	107 (35.4%) 27 (8.9%) 168 (55.6%) 36 (11.9%) 15 (5.0%) 31 (10.3%) 1 (0.3%)		Nutrition health risk level	Good (0–2 score) Moderate risk (3–5 score) High risk (6 or more)	
			
			
			
Living	Couples Relative or children Alone Others	130 (43.0%) 203 (67.3%) 44 (14.6%) 3 (1.0%)				
			
			
			

**Table 2:** Nutritional health risk indicators among study participants (*n* = 302).

Item	Frequency	Percent
I have an illness or condition that made me change type and/or amount of food I eat	136	45%
2. I eat fewer than 2 meals per day	23	7.6%
3. I eat few fruits, vegetables, or products of milk.	100	33.1%
4. I drink beer, liquor, or wine three times (a day?) almost every day.	8	2.6%
5. I have tooth and mouth problems that makes it difficult to eat.	139	46%
6. I do not always have enough money to buy the food I need.	64	21.2%
7. I eat alone most of the time.	157	52%
8. I take 3 or more prescribed or over-the-counter drugs a day.	123	40.7%
9. Without need, I lose or gain 10 pounds within 6 months.	28	9.3%
10. I am not always physically able to shop, cook, and/or nourish myself.	25	8.3%

**Table 3:** Mean and standard deviation (SD) of key variables among study participants (*n* = 302).

Variables	Mean ± SD
Cognitive ability (MMSE)	25.40 ± 0.219
Health Literacy	4.56 ± 0.92
SEPAS	7.52 ± 1.53
Food Security	4.84 ± 0.204

**Table 4:** Correlation between cognitive ability, physical activity self-efficacy, health literacy, and food security risk among older adults (*n* = 302).

	Cognitive Ability	Physical Activity Self-Efficacy	Health Literacy	Food Security Risk
Cognitive Ability	1	0.186 **	0.124 *	−0.137 *
Physical Activity Self-Efficacy	0.186 **	1	0.219 **	−0.237 *
Health Literacy	0.124 *	0.219 *	1	−0.154 *
Food Security Risk	−0.137 *	−0.237 *	−0.154 *	1

Statistical significance is indicated by asterisks: *p* < 0.01 (**) and *p* < 0.05 (*). The Pearson correlation test was employed to assess the relationships between these variables.

## Data Availability

The raw data supporting the conclusions of this article will be made available by the authors on request.
